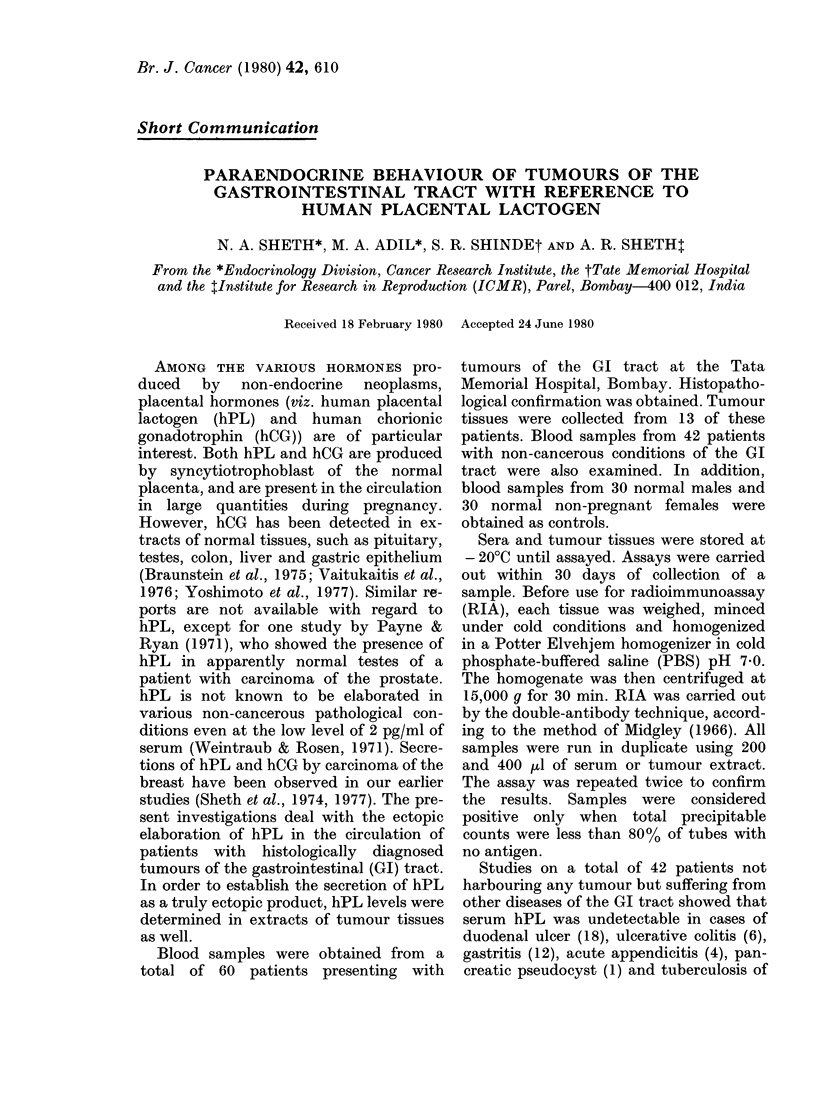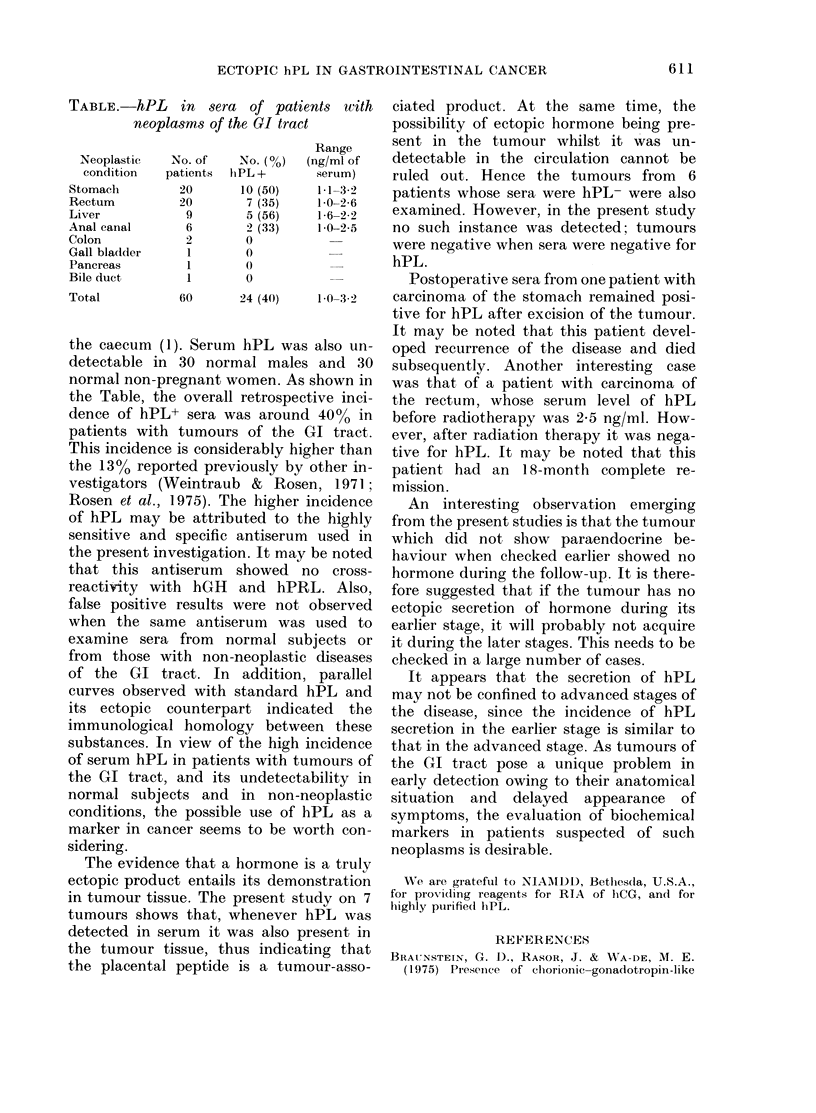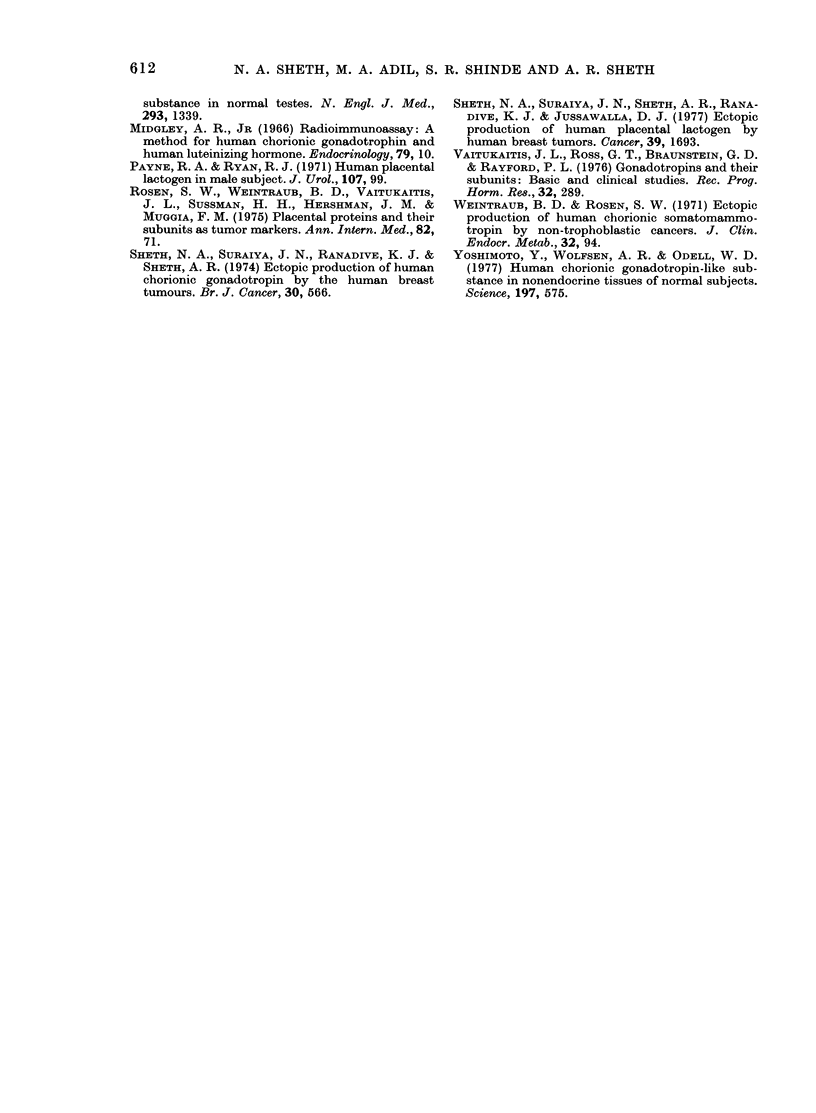# Paraendocrine behaviour of tumours of the gastrointestinal tract with reference to human placental lactogen.

**DOI:** 10.1038/bjc.1980.285

**Published:** 1980-10

**Authors:** N. A. Sheth, M. A. Adil, S. R. Shinde, A. R. Sheth


*Br. J. Cancer* (1980) **42**, 610

***Short Communication***

### PARAENDOCRINE BEHAVIOUR OF TUMOURS OF THE GASTROINTESTINAL TRACT WITH REFERENCE TO HUMAN PLACENTAL LACTOGEN

N. A. SHETH*, M. A. ADIL*, S. R. SHINDE† AND A. R. SHETH‡

*From the *Endocrinology Division, Cancer Research Institute, the †Tate Memorial Hospital and the ‡Institute for Research in Reproduction (ICMR), Parel, Bombay*—400 012, *India*

Received 18 February 1980 Accepted 24 June 1980

AMONG THE VARIOUS HORMONES produced by non-endocrine neoplasms, placental hormones (*viz.* human placental lactogen (hPL) and human chorionic gonadotrophin (hCG)) are of particular interest. Both hPL and hCG are produced by syncytiotrophoblast of the normal placenta, and are present in the circulation in large quantities during pregnancy. However, hCG has been detected in extracts of normal tissues, such as pituitary, testes, colon, liver and gastric epithelium (Braunstein *et al.*, 1975; Vaitukaitis *et al.*, 1976; Yoshimoto *et al.*, 1977). Similar reports are not available with regard to hPL, except for one study by Payne & Ryan (1971), who showed the presence of hPL in apparently normal testes of a patient with carcinoma of the prostate. hPL is not known to be elaborated in various non-cancerous pathological conditions even at the low level of 2 pg/ml of serum (Weintraub & Rosen, 1971). Secretions of hPL and hCG by carcinoma of the breast have been observed in our earlier studies (Sheth *et al.*, 1974, 1977). The present investigations deal with the ectopic elaboration of hPL in the circulation of patients with histologically diagnosed tumours of the gastrointestinal (GI) tract. In order to establish the secretion of hPL as a truly ectopic product, hPL levels were determined in extracts of tumour tissues as well.

Blood samples were obtained from a total of 60 patients presenting with tumours of the GI tract at the Tata Memorial Hospital, Bombay. Histopathological confirmation was obtained. Tumour tissues were collected from 13 of these patients. Blood samples from 42 patients with non-cancerous conditions of the GI tract were also examined. In addition, blood samples from 30 normal males and 30 normal non-pregnant females were obtained as controls.

Sera and tumour tissues were stored at −20°C until assayed. Assays were carried out within 30 days of collection of a sample. Before use for radioimmunoassay (RIA), each tissue was weighed, minced under cold conditions and homogenized in a Potter Elvehjem homogenizer in cold phosphate-buffered saline (PBS) pH 7·0. The homogenate was then centrifuged at 15,000 *g* for 30 min. RIA was carried out by the double-antibody technique, according to the method of Midgley (1966). All samples were run in duplicate using 200 and 400 $\mu$l of serum or tumour extract. The assay was repeated twice to confirm the results. Samples were considered positive only when total precipitable counts were less than 80% of tubes with no antigen.

Studies on a total of 42 patients not harbouring any tumour but suffering from other diseases of the GI tract showed that serum hPL was undetectable in cases of duodenal ulcer (18), ulcerative colitis (6), gastritis (12), acute appendicitis (4), pancreatic pseudocyst (1) and tuberculosis of

TABLE.—*hPL in sera of patients with neoplasms of the GI tract*

| Neoplastic condition | No. of patients | No. (%) hPL+ | Range (ng/ml of serum) |
|---|---|---|---|
| Stomach | 20 | 10 (50) | 1·1–3·2 |
| Rectum | 20 | 7 (35) | 1·0–2·6 |
| Liver | 9 | 5 (56) | 1·6–2·2 |
| Anal canal | 6 | 2 (33) | 1·0–2·5 |
| Colon | 2 | 0 | — |
| Gall bladder | 1 | 0 | — |
| Pancreas | 1 | 0 | — |
| Bile duct | 1 | 0 | — |
| Total | 60 | 24 (40) | 1·0–3·2 |

the caecum (1). Serum hPL was also undetectable in 30 normal males and 30 normal non-pregnant women. As shown in the Table, the overall retrospective incidence of $hPL^+$ sera was around 40% in patients with tumours of the GI tract. This incidence is considerably higher than the 13% reported previously by other investigators (Weintraub & Rosen, 1971; Rosen *et al.*, 1975). The higher incidence of hPL may be attributed to the highly sensitive and specific antiserum used in the present investigation. It may be noted that this antiserum showed no cross-reactivity with hGH and hPRL. Also, false positive results were not observed when the same antiserum was used to examine sera from normal subjects or from those with non-neoplastic diseases of the GI tract. In addition, parallel curves observed with standard hPL and its ectopic counterpart indicated the immunological homology between these substances. In view of the high incidence of serum hPL in patients with tumours of the GI tract, and its undetectability in normal subjects and in non-neoplastic conditions, the possible use of hPL as a marker in cancer seems to be worth considering.

The evidence that a hormone is a truly ectopic product entails its demonstration in tumour tissue. The present study on 7 tumours shows that, whenever hPL was detected in serum it was also present in the tumour tissue, thus indicating that the placental peptide is a tumour-associated product. At the same time, the possibility of ectopic hormone being present in the tumour whilst it was undetectable in the circulation cannot be ruled out. Hence the tumours from 6 patients whose sera were $hPL^-$ were also examined. However, in the present study no such instance was detected; tumours were negative when sera were negative for hPL.

Postoperative sera from one patient with carcinoma of the stomach remained positive for hPL after excision of the tumour. It may be noted that this patient developed recurrence of the disease and died subsequently. Another interesting case was that of a patient with carcinoma of the rectum, whose serum level of hPL before radiotherapy was 2·5 ng/ml. However, after radiation therapy it was negative for hPL. It may be noted that this patient had an 18-month complete remission.

An interesting observation emerging from the present studies is that the tumour which did not show paraendocrine behaviour when checked earlier showed no hormone during the follow-up. It is therefore suggested that if the tumour has no ectopic secretion of hormone during its earlier stage, it will probably not acquire it during the later stages. This needs to be checked in a large number of cases.

It appears that the secretion of hPL may not be confined to advanced stages of the disease, since the incidence of hPL secretion in the earlier stage is similar to that in the advanced stage. As tumours of the GI tract pose a unique problem in early detection owing to their anatomical situation and delayed appearance of symptoms, the evaluation of biochemical markers in patients suspected of such neoplasms is desirable.

We are grateful to NIAMDD, Bethesda, U.S.A., for providing reagents for RIA of hCG, and for highly purified hPL.

### REFERENCES

BRAUNSTEIN, G. D., RASOR, J. & WA-DE, M. E. (1975) Presence of chorionic–gonadotropin-like substance in normal testes. *N. Engl. J. Med.*, **293**, 1339.

MIDGLEY, A. R., JR (1966) Radioimmunoassay: A method for human chorionic gonadotrophin and human luteinizing hormone. *Endocrinology*, **79**, 10.

PAYNE, R. A. & RYAN, R. J. (1971) Human placental lactogen in male subject. *J. Urol.*, **107**, 99.

ROSEN, S. W., WEINTRAUB, B. D., VAITUKAITIS, J. L., SUSSMAN, H. H., HERSHMAN, J. M. & MUGGIA, F. M. (1975) Placental proteins and their subunits as tumor markers. *Ann. Intern. Med.*, **82**, 71.

SHETH, N. A., SURAIYA, J. N., RANADIVE, K. J. & SHETH, A. R. (1974) Ectopic production of human chorionic gonadotropin by the human breast tumours. *Br. J. Cancer*, **30**, 566.

SHETH, N. A., SURAIYA, J. N., SHETH, A. R., RANADIVE, K. J. & JUSSAWALLA, D. J. (1977) Ectopic production of human placental lactogen by human breast tumors. *Cancer*, **39**, 1693.

VAITUKAITIS, J. L., ROSS, G. T., BRAUNSTEIN, G. D. & RAYFORD, P. L. (1976) Gonadotropins and their subunits: Basic and clinical studies. *Rec. Prog. Horm. Res.*, **32**, 289.

WEINTRAUB, B. D. & ROSEN, S. W. (1971) Ectopic production of human chorionic somatomammotropin by non-trophoblastic cancers. *J. Clin. Endocr. Metab.*, **32**, 94.

YOSHIMOTO, Y., WOLFSEN, A. R. & ODELL, W. D. (1977) Human chorionic gonadotropin-like substance in nonendocrine tissues of normal subjects. *Science*, **197**, 575.